# An economon model of drug addiction

**DOI:** 10.1007/s00213-024-06535-7

**Published:** 2024-01-26

**Authors:** S. Stevens Negus

**Affiliations:** Department of Pharmacology and Toxicology, Virginia Commonwealth University, 410 N. 12 St, Richmond, VA 23298 USA

**Keywords:** Economy, Addiction, Model, Operant behavior

## Abstract

The term “economon” (i:’ka.nə.muhn; plural: economa) is introduced here to describe an economic unit composed of two participants engaged in mutually reinforcing operant behavior. Economa are basic building blocks of transactional behavior that aggregate in social networks called economies. In a drug-addiction economon, operant behavior by one participant (the “supplier”) provides an addictive drug as a reinforcer to the second participant (a “Person with Substance Use Disorder; PwSUD”). Reciprocal operant behavior by the PwSUD usually provides money as a reinforcer to the supplier. After defining the features of the drug-addiction economon, this article discusses its implications for (1) prevalence and virulence of drug addiction, (2) opportunities for drug-addiction research in general, (3) the “brain-disease model of addiction” in particular, and (4) factors that mitigate harm or promote risk of drug addiction. The economon model is intended to provide a novel perspective on the uniquely human disorder of drug addiction.

## Introduction

**Fig. 1:**
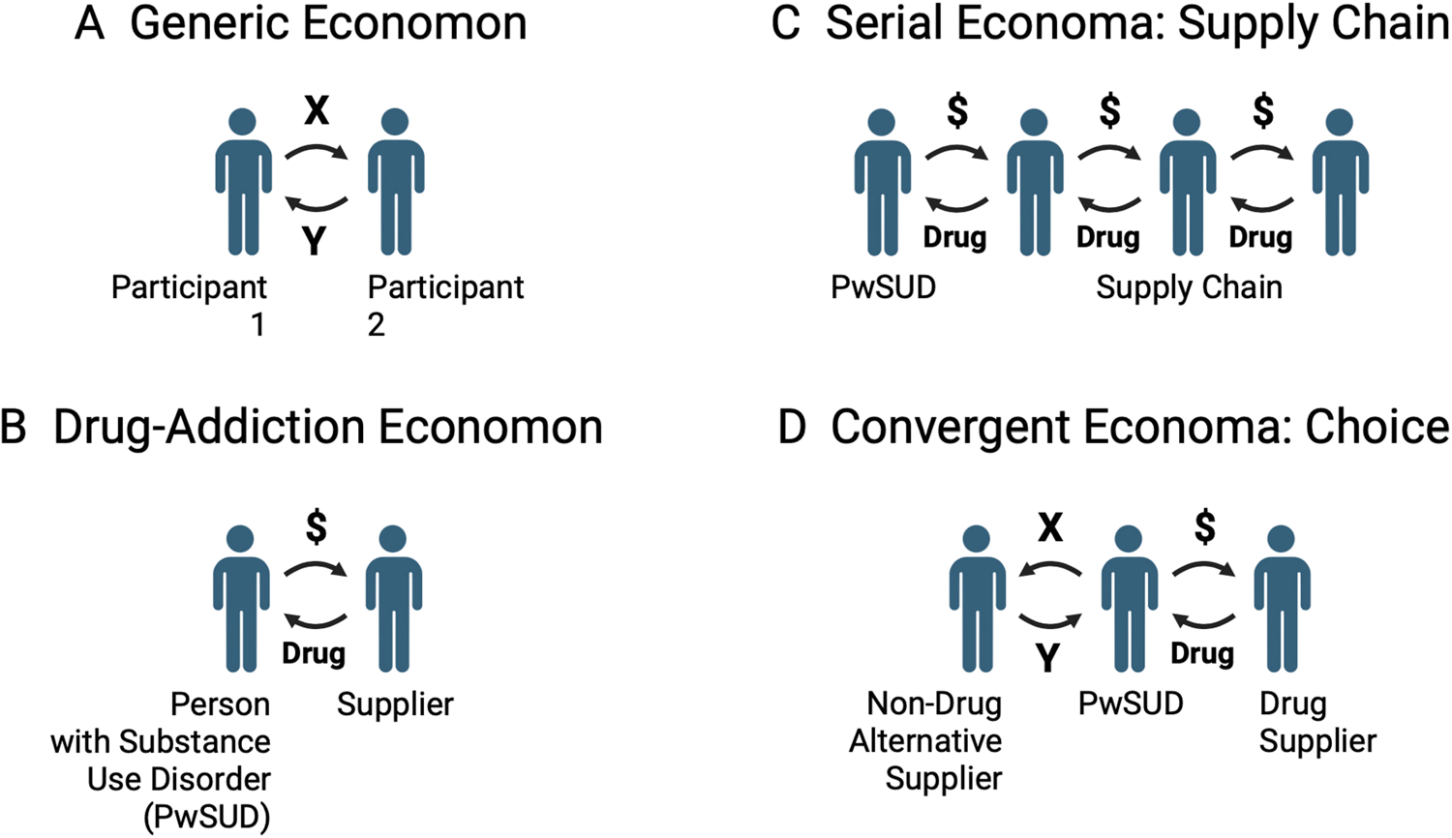
Overview of the economon model of drug addiction. **A** An economon is an economic unit composed of two participants engaged in mutually reinforcing behavior. The participants serve as discriminative stimuli for each other. Operant behavior emitted by participant 1 produces a stimulus *X* that functions as reinforcer in participant 2, while reciprocal operant behavior emitted by participant 2 produces a stimulus *Y* that functions as a reinforcer in participant 1. **B** In a drug-addiction economon, an addictive drug such as fentanyl or cocaine serves as the reinforcer for a “person with substance use disorder (PwSUD)” while money usually serves as the reinforcer for a “supplier.” Drug-addiction economa are resilient because consumption of both addictive drugs and money is resistant to satiation. **C** Drug-addiction economa can be organized in series to form supply chains. **D** Drug supply chains can converge with supply chains for alternative reinforcers at the level of the PwSUD. The PwSUD allocates his or her behavior between the two suppliers in the process of “choice.” Created with Biorender

Substance use disorders are a uniquely human and relatively modern class of behavioral disorders responsible for an escalating rate of overdose deaths. This article introduces a new model to frame key components of drug addiction and guide strategies for its mitigation. The article is divided into two main sections. First, drug addiction is characterized as a pathology rooted in learned and mutually reinforcing operant behavior emitted by two participants: (1) a person with substance use disorder (PwSUD), who learns to acquire and consume the drug, and (2) a supplier, who learns to produce and distribute the drug. Together, these two participants form an interlocking economic unit that will be referred to here by the term “economon” (i:’ka.nə.muhn; plural: economa). Figure 1 illustrates the structure of an individual economon and its role as a building block of broader social networks that form economies. The second part of the article will discuss some implications of this economon model.

## The economon model of drug addiction

Drug use is a learned behavior acquired through operant conditioning, which associates behavior with its consequences (Kelleher and Goldberg 1975; Negus and Banks 2011; Skinner 1938). The essential components of operant conditioning are expressed by the “three-term contingency” diagrammed as

$${S}^{D}\to R\to {S}^{C}$$

where *R* denotes a “response” (or behavior) emitted by the subject, *S*^*C*^ denotes a “consequent stimulus” delivered back to the subject as a consequence of its behavior, and *S*^*D*^ denotes a “discriminative stimulus” that signals the probability that performance of *R* will result in receipt of *S*^*C*^. The consequent stimulus *S*^*C*^ can be further categorized according to its impact on the probability of behaviors that precede its delivery. A “reinforcing stimulus” produces “reinforcing effects” to increase probability of preceding behavior, whereas a “punishing stimulus” produces “punishing effects” to decrease probability of the preceding behavior. Many addictive drugs produce reinforcing effects that can be studied in drug self-administration procedures distinguished by a simplified arrangement of these three terms. For example, a rat with an intravenous catheter could be placed into an experimental chamber equipped with a stimulus light, a response lever, and a syringe pump filled with a fentanyl solution and connected to the catheter. Contingencies can be programmed by the investigator such that illumination of the stimulus light serves as a discriminative stimulus (*S*^*D*^) to signal that lever-pressing behavior (*R*) by the rat will result in the consequence of pump activation and intravenous fentanyl delivery (the *S*^*C*^). Conversely, when the stimulus light is off, then lever pressing does not lead to pump activation and fentanyl delivery. After repeated exposure to these conditions to enable operant learning, most rats will emit higher rates of lever pressing when the light is on and responding produces drug than when it is off and responding does not produce drug, providing one source of evidence that the drug functions as a reinforcer. In human drug addiction, the nature of the *S*^*D*^, *R*, and *S*^*C*^ is more complex, but the same principles apply.

Drug reinforcement is a key driver of drug consumption and ultimately contributes to severe SUD and addiction; however, drug reinforcement is not sufficient. Drug addiction also depends on drug supply. Under the rubric of operant conditioning and the three-term contingency, it is theoretically possible for the behavior *R* of a single individual to encompass all the activities required to produce and consume a sustained drug supply, but this is exceedingly rare. Most addictive drugs have agricultural origins (e.g., cocaine from coca, morphine from poppies, nicotine from tobacco, alcohol from fermentation of a range of carbohydrate sources). These plant-based raw materials are geographically and seasonally limited and require a suite of learned behaviors to cultivate, harvest, and prepare the plant and its products for ingestion. More recently developed synthetic drugs (e.g., fentanyl, methamphetamine) can be produced with less effort in a modestly equipped laboratory, but it remains necessary to secure the skills, equipment, and raw materials. In either case, substantial, sophisticated, and sustained expression of learned, operant behavior is required to produce a drug supply sufficient to fuel drug addiction. For non-human animals, and for humans during most of our evolutionary history, the cognitive and technical demands of drug production have served as a constraint on drug supply and an obstacle to drug consumption and addiction. However, the emergence and evolution of economa in human culture provide a mechanism to surmount this obstacle by enabling production and consumption to be emitted as two mutually reinforced behaviors by two participants.

The economon is a general unit of transactional behavior in which two organisms engage in mutually reinforcing behavior as defined by the three-term contingency. In an economon, each participant serves as a source of discriminative stimuli for operant behavior by the other participant. In the context of that discriminative stimulus, each participant is increasingly likely to emit behavior *R* that results in production and delivery of a reinforcing stimulus *S*^*R*^ to the other. The behaviors emitted and stimuli exchanged in an economon can assume a wide variety of forms, with the behaviors emitted often described by terms such as “work” (by the supplier) and “consumption” (by the consumer), and the stimuli exchanged described as “goods” or “services.” Two examples illustrate the expression of this mutually reinforced behavior when one of the reinforcing stimuli is an addictive drug. First, the account provided above for fentanyl self-administration by a rat can be expanded to include the investigator as the second participant. Laboratory work by the investigator makes fentanyl available as a reinforcing stimulus for consumption by the rat, while lever-pressing behavior by the rat makes data available as a reinforcing stimulus to the investigator. Second, in a case of clinical drug addiction, the PwSUD and drug supplier are also linked in a relationship of intermittent and mutually reinforcing behavior. The supplier emits behavior to make drug available as a reinforcing stimulus for consumption by the PwSUD, while the PwSUD emits behavior to provide a stimulus, usually money, sufficient to serve as a reinforcer to the supplier. In summary, the fundamental feature of any economon is a relationship of mutually reinforced behavior by two participants (Fig. 1A). The drug-addiction economon is a subtype in which an addictive drug serves as the principal reinforcer for one participant, and drug consumption causes harm in that participant (Fig. 1B).

Economa serve as the fundamental economic unit of all trade, and in humans, economa assemble into social networks that form economies. By analogy to neural circuits composed of neurons, these economic networks can arrange economa in serial or in convergent/divergent circuits. Thus, for any one commodity, a series of economa can form a supply chain that ultimately delivers the commodity to the end consumer (Fig. 1C). For any pair of commodities, two different suppliers can converge on a consumer whose behavior diverges across the suppliers (Fig. 1D). In this latter case, the behavior emitted by the suppliers competes for the behavior and associated reinforcers emitted by the consumer, and the consumer reciprocally allocates his or her behavior between the two suppliers in a process that can be called “choice.” Both competition by the suppliers and choice by the consumer are dynamic, and to the degree enabled by the suppliers, choice by the end user typically alternates between different suppliers of different commodities in a manner that yields a mix of reinforcers sufficient to sustain consumer health. In particular, the process of satiation during serial consumption serves to reduce effectiveness of many reinforcers, limit the duration and frequency of economon operation, and facilitate consumer switching between suppliers of different commodities. However, the drug-addiction economon is unusually resilient due to the reinforcers that sustain it. The supplier is typically reinforced by money, which has evolved to function as a generic social commodity in human economies that is both broadly valued and resistant to satiation as a constraint on its consumption (Easterlin 1974; Jebb et al. 2018). The PwSUD is reinforced by an addictive drug that pharmacologically activates core neurobiological mechanisms of reinforcement and is also resistant to satiation (Aigner and Balster 1978; Deneau et al. 1969). Initial exposure to these reinforcers has the potential to drive operant learning and progressively shape behavior as with the operation of any schedule of reinforcement. However, unfettered by satiation, the reciprocal positive-feedback loop of behaviors emitted by participants in a drug-addiction economon can accelerate in frequency to the increasing exclusion of other behaviors. The conventional definition of drug addiction focuses on the behavior and health of the PwSUD, and a key clinical sign of severe SUD is excessive allocation of behavior to drug use (i.e., excessive drug choice) at the expense of more adaptive behaviors maintained by alternative reinforcers (Banks and Negus 2017; Heyman 2010). Reciprocally, the drug supplier may also be “addicted”; in the supplier’s case, that addiction manifests as excessive allocation of behavior to drug supply.

## Implications of the economon model of drug addiction

### Prevalence and virulence.

Once established, a drug-addiction economon is resilient because it is fueled by money and an addictive drug as two reciprocally delivered reinforcers that are resistant to satiation and hence persistent in their reinforcing efficacy. However, drug-addiction economa pose a threat to human health not only because they are individually resilient, but also because they can spread. Within an individual, the consequence of operant reinforcement is the replication of a reinforced behavior. The economon provides a mechanism for sustained replication of two mutually reinforcing behaviors by the two participants. These replicating behaviors in one economon can then be mimicked by or taught to other persons and thereby transmitted from one person to another to generate new economa. In this regard, economa in general can be viewed as social vehicles for mutually reinforcing human behavioral “replicators,” or memes, that are analogous to the function of biological organisms as vehicles for genes as biochemical replicators (Blackmore 1999; Dawkins 1976). Moreover, as with genes, the behaviors embedded in economa can vary and mutate across time, compete with other economa, and evolve in response to environmental selection pressures such as changing regulations and enforcement strategies.

**Fig. 2:**
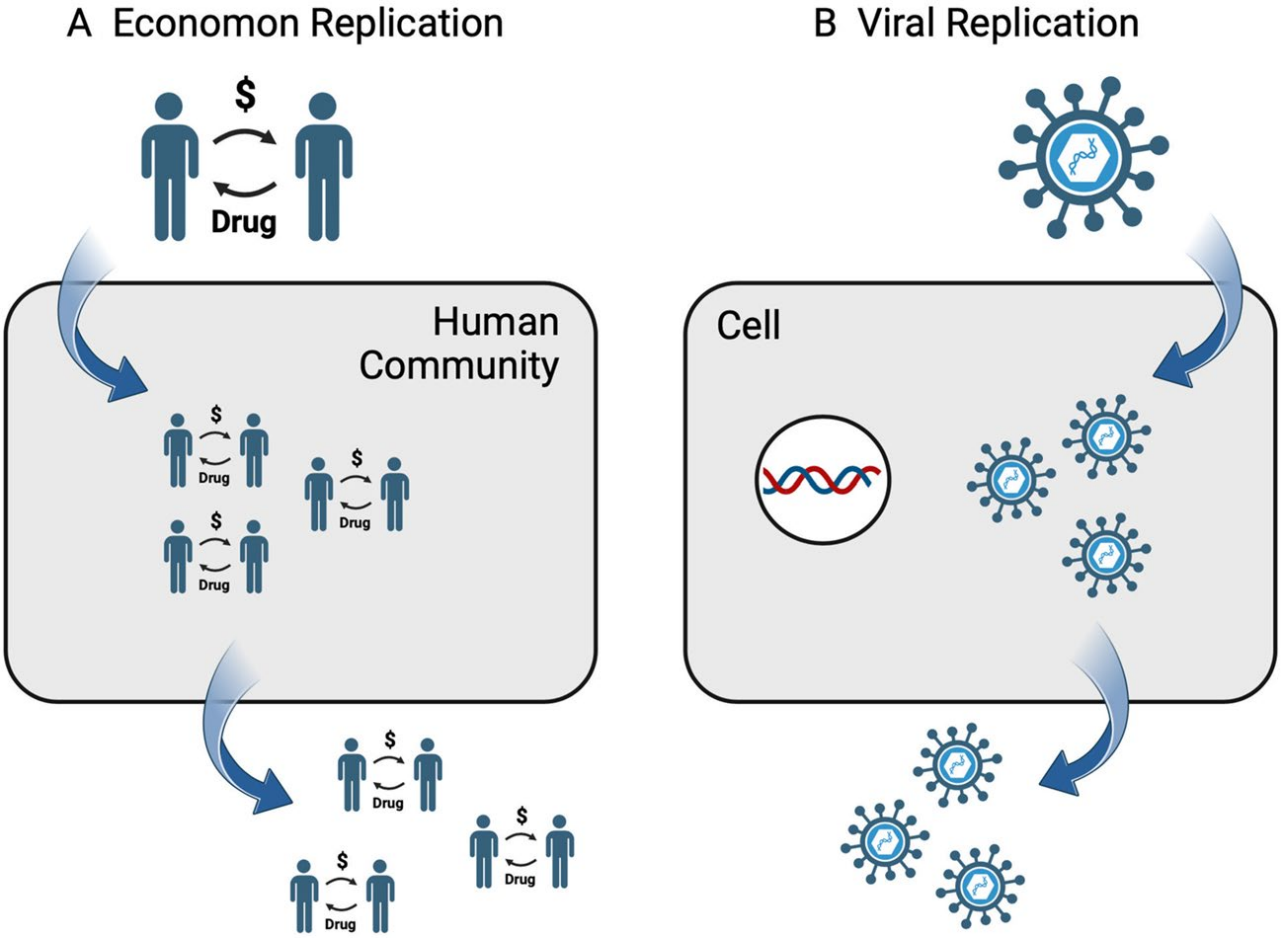
Drug-addiction economa and viruses as parasitic replicators. **A** Drug-addiction economa contain reciprocal social behaviors (memes) that can enter a human community and exploit communal resources and the operant behavioral processes of its human residents to replicate within that community. These economa can then leave the initial host community and infect other communities. **B** Viruses contain genes that can enter a cell and exploit cellular resources and the processes of transcription and translation to replicate within that cell. Viral particles can then leave the initial host cell to infect other cells. Created with Biorender

Many economa facilitate the exchange of vital commodities and function as symbiotic constituents of the human communities that host them. Drug-addiction economa, by contrast, function as “parasitic replicators” that achieve their own replication at the expense of the host. The prototype parasitic replicator is a virus, which commandeers biochemical processes of transcription and translation in a cell to replicate virus, often at the expense of the health or life of the infected cell (Koonin and Starokadomskyy 2016). Drug-addiction economa consist of a collection of behaviors, or memes, rather than of genes, and they operate at the level of human communities rather than at the level of a single cell. However, as illustrated in Fig. 2, the principles of replication are analogous. As a drug-addiction economon enters a human community, it commandeers operant-behavioral processes that underlie normal economic interaction to replicate drug-addiction economa. The proliferation of drug-addiction economa harms community health both by displacing healthy economa and by producing toxic effects in PwSUD. Moreover, new drug-addiction economa can then leave an initial host to target new communities.

The identity of drug-addiction economa as parasitic replicators suggests that, as for any such replicator, both prevalence and virulence will evolve in response to environmental selection pressures (Cressler et al. 2016; Ewald 2004). On the one hand, the behaviors for both drug supply and drug use can be expected to evolve in ways that increase mutual reinforcement to the supplier and consumer, increase reproductive success of the economon, and increase prevalence. On the other hand, virulence expressed in particular as high acute toxicity can be a constraint on prevalence insofar as it harms the host; however, this constraint can be evaded so long as a parasitic replicator can transfer to new hosts once an existing host has been depleted. Accordingly, high virulence may be tolerated until it impedes replication. The trajectories of alcohol and opioid use over time and across different human cultures may illustrate different evolutionary stages that can occur as drug-use economa adapt to their human communal hosts. Alcohol-use economa have evolved over centuries in Western society to reach a relatively high and stable prevalence with clinically significant but limited acute toxicity. By contrast, the ongoing epidemic of opioid use and overdose deaths in the USA illustrates a recent period of rapid evolution in opioid-use economa that continues to exhibit high virulence.

### Drug-addiction research.

**Fig. 3:**
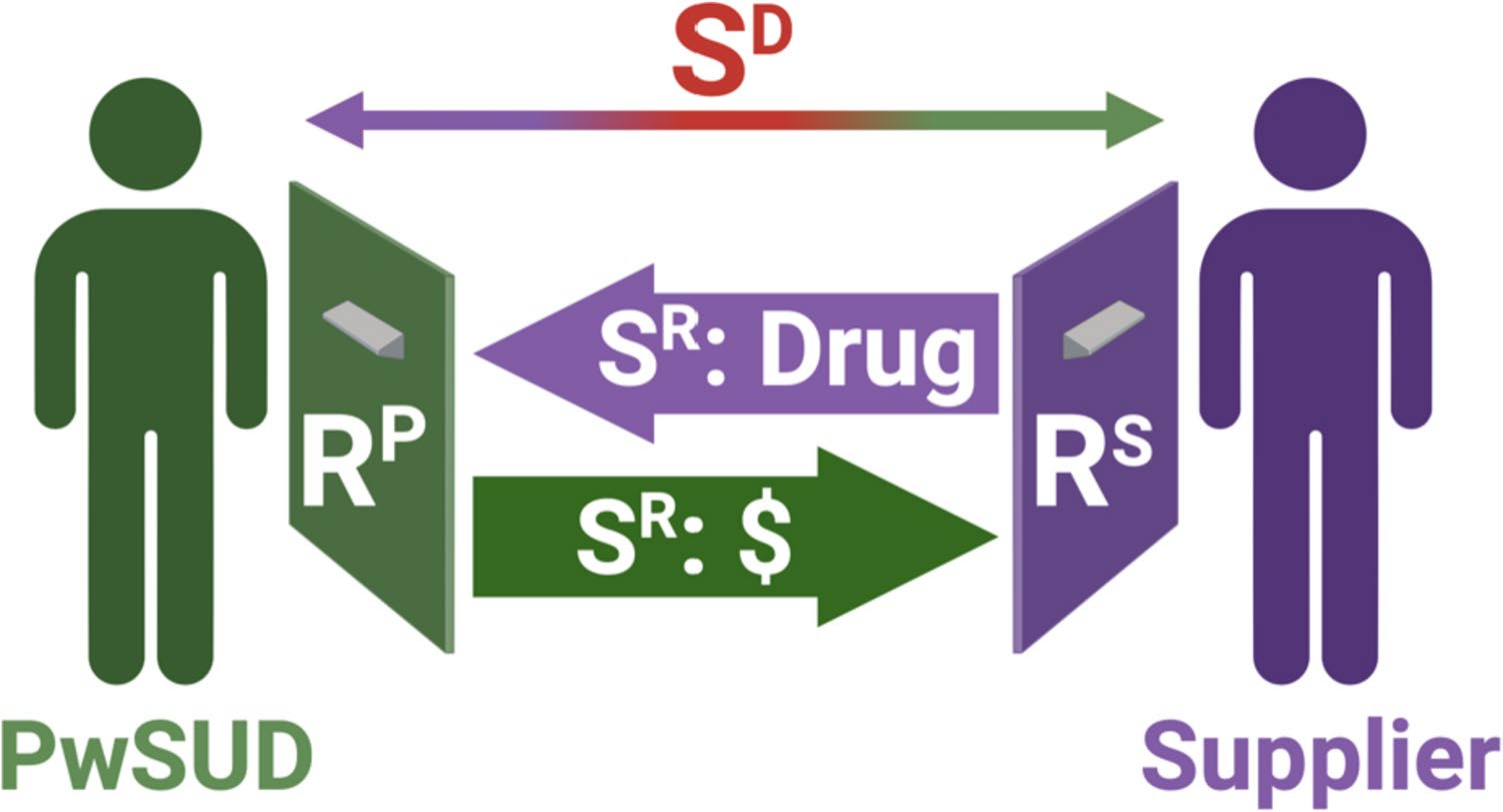
A laboratory-based arrangement for research on drug-addiction economa. In a human-laboratory context, two participants could each be given access to a response lever that activates reinforcer delivery to the other participant. The PwSUD and supplier serve as discriminative stimuli (*S*^*D*^) to set the occasion for each other’s behavior. In the presence of this discriminative stimulus, responding by the supplier (*R*^*S*^) makes drug available to the PwSUD (*S*^*R*^: drug). Reciprocally, responding by the PwSUD (*R*^*P*^) delivers money as a reinforcing stimulus to the supplier (*S*^*R*^: $). Variables amenable to manipulation include the magnitude of reinforcers, the schedules of reinforcement for both consumer and supplier, and the presence or absence of alternative reinforcers, punishers, or candidate treatments. Created with Biorender

Drug-addiction economa can provide a novel topic for both laboratory and field research. In the laboratory, a simple economon could be established as illustrated in Fig. 3 using human subjects in two interacting operant-conditioning systems, each equipped with a manipulandum such as a response lever. At the onset of an experimental trial, the two subjects would serve as discriminative stimuli (*S*^*D*^) for each other, either through direct visual contact or some shared stimulus such as a light to indicate each other’s presence. Responses by the supplier (*R*^*Supplier*^) would make drug available to the PwSUD for some limited period of time (e.g., 1 min). Responses by the PwSUD (*R*^*PwSUD*^) during this period of drug availability would then activate drug delivery as the reinforcing stimulus to the PwSUD (*S*^*R*−*PwSUD*^) while also delivering money as the reinforcing stimulus to the supplier (*S*^*R*−*Supplier*^). This basic set of contingencies might be expected to engender a reverberating cycle of reciprocal responding by supplier and PwSUD. Next, imagine treating the PwSUD with an effective opioid-use-disorder medication such as buprenorphine. This would be expected to reduce PwSUD responding and fentanyl consumption, but it would also reduce supplier reinforcement and might trigger compensatory supplier behavior aimed at re-engaging the PwSUD (e.g., more frequent responding by the supplier to produce more frequent drug availability to the PwSUD). This type of compensatory supplier behavior is a prominent but understudied barrier to treatment of the PwSUD.

Laboratory-based economa can also be established with laboratory animals, but it would of course be necessary to use a reinforcer other than money for the “supplier.” Insofar as satiation-resistance is an important feature of the reinforcers in drug-addiction economa, it might be advantageous to select a satiation-resistant reinforcer, such as electrical brain stimulation (Olds 1958) or drug delivery, for the subject in the supplier role.

The components and operation of drug-addiction economa can also be investigated in the field by considering the reciprocal behavior of both PwSUD and suppliers in clinical drug-addiction relationships, and this research is most commonly conducted and published within the academic discipline of economics. Drug-addiction economa will be easiest to observe for alcohol and tobacco products because these are licit drugs with both identifiable consumers and regulated and observable suppliers (e.g. Cook and Moore 2002). As will be noted further below, agonist medications for treatment of drug addiction (i.e., methadone and buprenorphine for opioid use disorder) can also be conceptualized as drug reinforcers delivered to identifiable PwSUD by regulated and observable suppliers (e.g., Murphy and Polsky 2016). Illicit drug markets will be more difficult to study given their operation outside of regulatory frameworks, but they can still be investigated using approaches that include targeted interviews and consideration of law-enforcement and medical data (e.g., Caulkins et al. 1999, 2006; Caulkins and Reuter 2006). Across drug types and regulatory contexts, economic principles can yield insights into both the drug-consumption behavior by the PwSUD (Bickel et al. 2014) and the entrepreneurial behavior of suppliers (March et al. 2016).

### Brain disease model of addiction.

The “brain-disease model of addiction” is a prominent contemporary framework for drug-addiction research that focuses on the brain of the PwSUD (Koob 2021; Volkow and Koob 2015). This model posits that repeated drug exposure produces cumulative and lasting changes in PwSUD brain structure and function to increase net drug reinforcement and drive escalating drug consumption. The present economon model does not exclude processes described by the brain-disease model of addiction, but neither does it require them. Both participants in a drug-addiction economon require a nervous system to detect stimuli, emit behaviors, and learn associations that link stimuli and behaviors. For the PwSUD, drug reinforcement requires drug molecules to interact with target receptors in the brain and produce a net stimulation of brain reinforcement systems. Stimulation of mesolimbic dopamine signaling in particular functions as a final common pathway for neural encoding of behavioral reinforcement by many natural reinforcers (food, sex) and addictive drugs (Ikemoto and Bonci 2014). However, natural reinforcers activate this system indirectly via multi-neuronal circuits that originate in the periphery, project centrally, and habituate during repeated stimulation to produce the behavioral process of satiation (Amin and Mercer 2016; Levin 2009; Ritter 2004). Addictive drugs, by contrast, bind to central nervous system receptors to activate the mesolimbic dopamine system more directly via processes that are relatively resistant to satiation (Koob 1992). This effectiveness of addictive drugs to produce satiation-resistant stimulation of mesolimbic dopamine release is sufficient to promote continued drug-taking behavior that could ultimately meet criteria for a substance use disorder, especially in environmental contexts where drug supply is abundant and cheap while alternative reinforcers are scarce or expensive.

Drug-induced changes in the brain of the PwSUD that increase drug reinforcement as proposed by the brain-disease model of addiction could certainly contribute to a drug-addiction economon. However, the Economon Model of drug-addiction proposed here also implies a broader range of mechanisms that could reside not only in the brain of the PwSUD, but also in the brain of the supplier and in the economic environment inhabited by both participants. One particular neurobiological mechanism in the brain of the PwSUD warrants brief consideration here as a corollary to current brain-disease models. The Economon Model places the PwSUD at the nexus of competing commodity supply chains, a subset of which can supply drugs. The PwSUD allocates his or her behavior, or “chooses,” between the available suppliers and their commodities, and behavioral allocation to drug choice will be influenced not only by the absolute reinforcing effects of the drug, but also by the relative strength of drug reinforcement in comparison to reinforcement by non-drug alternatives. Accordingly, chronic drug exposure could *increase* drug choice by *decreasing* the reinforcing effects of non-drug alternatives. Consistent with this possibility, it is well established that chronic drug exposure can produce dependence characterized by the emergence of a physiological and behavioral abstinence syndrome when drug is withdrawn, and one common component of withdrawal from many addictive drugs is a decrease in the reinforcing effectiveness of non-drug reinforcers such as food and social interaction (Emmett-Oglesby et al. 1990; Negus and Rice 2009; Spragg 1940). Accordingly, during drug withdrawal, relative drug reinforcement and resulting drug consumption may increase due not to an increase in drug reinforcement, but to a decline in reinforcing effects of alternatives. The impact of drug dependence and withdrawal on relative rather than absolute drug reinforcement has been emphasized previously in models that propose a cumulative *reduction* in reinforcing effects of both drugs and alternatives, with relative drug reinforcement and associated drug use increasing because drug reinforcement is reduced less than reinforcement by alternatives (Herrnstein and Prelec 1992; Rachlin 1997).

We have referred to this process as “heterologous reinforcer desensitization” to capture the idea that drug consumption can desensitize mechanisms that underlie reinforcement by non-drug alternatives and thereby decrease effectiveness of those alternatives to compete for allocation of behavior (Negus and Banks 2021). Additionally, in the context of the economon model of drug addiction, the process of heterologous reinforcer desensitization illustrates a more general strategy of host-repression in the action of successful parasitic replicators. Viruses, for example, rely on the biochemical functions of their cellular hosts to replicate viral nucleic acids and proteins. As such, the virus competes with the host cell for control of cellular transcriptional and translational machinery, and many viruses include genes for proteins that impede host-cell access to this machinery (Lyles 2000; Pardamean and Wu 2021). By analogy, natural selection may come to favor drug-addiction economa that supply drugs capable not only of producing direct reinforcement, but also of producing heterologous reinforcer desensitization as a mechanism for degrading sensitivity to alternative reinforcers.

### Mitigation and risk.

**Fig. 4:**
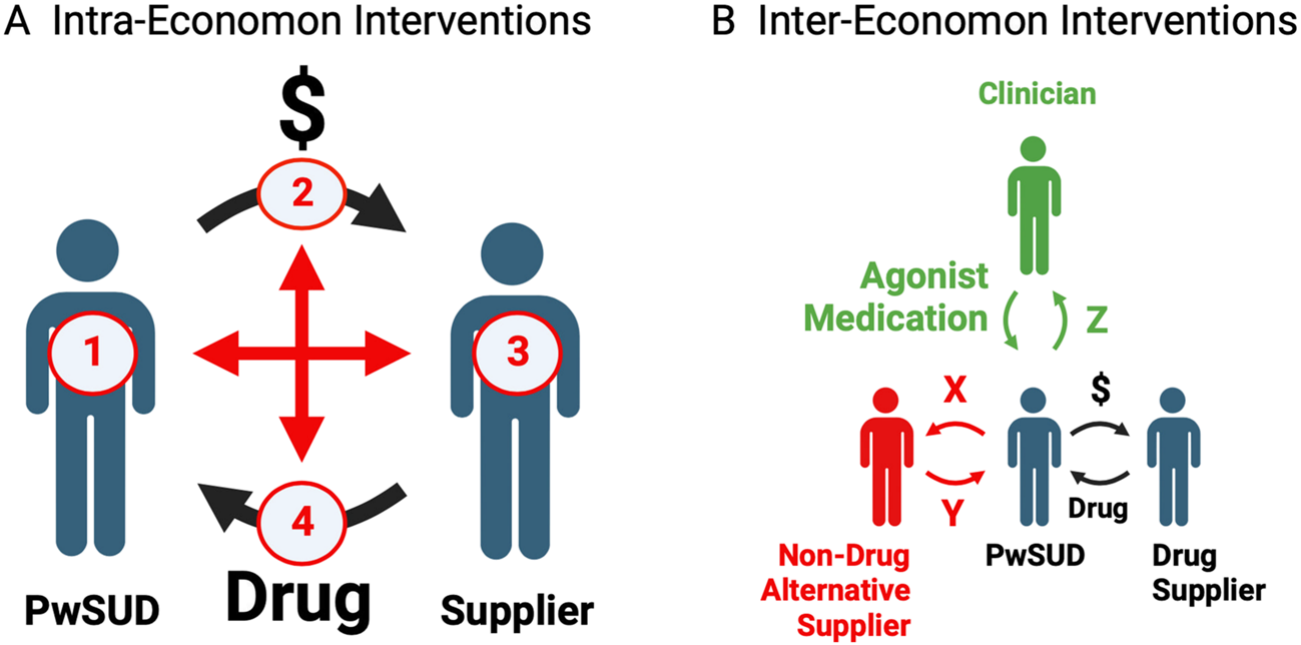
Strategies to mitigate the harm of drug-addiction economa. **A** Intra-economon interventions can target the internal cycle of a drug-addiction economon by attempting to (1) block PwSUD detection of the drug reinforcer, (2) block or punish PwSUD behavior that generates payment to the supplier, (3) block supplier detection of the monetary reinforcer, or (4) block or punish supplier behavior that generates drug supply to the PwSUD. **B** Inter-economon interventions can seek to amplify the impact of alternative economa that compete with the drug-addiction economon for allocation of PwSUD behavior. Alternative economa can offer supply of either non-drug reinforcers (food, social interaction; red) or agonist medications that are safer to the PwSUD than the addictive drug (e.g., buprenorphine or methadone for opioid use disorder; green). Created with Biorender

Drug-addiction economa harm the health of both the PwSUD and the community in which both PwSUD and supplier reside. Mitigation of that harm can be attempted by two general strategies illustrated in Fig. 4. First, intra-economon interventions can theoretically target each internal node of the operant-conditioning cycle at the heart of a drug-addiction economon: (1) block PwSUD detection of the reinforcing drug stimulus; (2) block or punish PwSUD behavior that generates payment to the supplier; (3) block supplier detection of the reinforcing monetary stimulus; (4) block or punish supplier behavior that generates drug supply to the PwSUD. In practice, these interventions are challenging or impossible to implement. PwSUD detection of the drug stimulus (Node 1) can be attenuated with medications that pharmacologically block receptors for some addictive drugs (e.g., naltrexone for opioid receptors that mediate opioid reinforcement), but compliance with antagonist medications is problematic (Blum et al. 2020; Townsend et al. 2021), and such medications are not available for most addictive drugs (e.g., for cocaine and methamphetamine that act as dopamine transporters). Vaccines represent an emerging set of medications that may also impede drug access to receptors that mediate reinforcement (Townsend and Banks 2020; Truong and Kosten 2022). In contrast to these marginally effective tools for blocking drug detection by the PwSUD, there are no available strategies for blocking detection of monetary reinforcement by the supplier (Node 3). Behavioral blockade (e.g., by imprisonment) or punishment (e.g., by fines) can be directed at the PwSUD (Node 2) or supplier (Node 4) and are widely used tools for combatting drug-addiction economa (Courtwright 2004; Csete et al. 2016; Davis et al. 2016); however, challenges with these tools include their expense, the vulnerability of physical blockade or punishment to evasion, the weak efficacy of punishment when its delivery is inconsistent or delayed, and the potential for unintended consequences such as transitions by supplier and PwSUD to more potent drugs.

Second, inter-economon interventions can focus on the external economic environment in which a drug-addiction economon operates, and in particular, on the influence of other supply chains that converge on the PwSUD and compete with drug for allocation of PwSUD behavior. The most common commodities delivered by competing supply chains are non-drug reinforcers such as food or social reinforcement, and a rich and reliable array of non-drug alternatives may be especially useful in discouraging entry of both consumers and suppliers into drug-addiction economa (Galaj et al. 2020; Heilig et al. 2016; Hsiung et al. 2022). However, as noted above, once a PwSUD does enter a drug-addiction economon, repeated drug consumption may produce heterologous reinforcer desensitization to reduce biological sensitivity to non-drug reinforcers, especially during periods of drug withdrawal. Additionally, blockade or punishment of behavior may further isolate a PwSUD and reduce access to non-drug alternatives. Consequently, once enmeshed in a drug-addiction economon, it may become more effective to use agonist-medication strategies (e.g., buprenorphine or methadone for opioid use disorder) to enable supply of a drug that is sufficiently effective as a reinforcer to compete with the addictive drug, safer than the addictive drug, and legally accepted (Grabowski et al. 2004; Townsend et al. 2021). Whether using non-drug alternatives or agonist medications, the deployment of alternative commodities via alternative supply chains will benefit from use of marketing strategies that optimize allocation of consumer behavior away from the addictive drug and toward healthier alternatives.

The foundation of drug-addiction economa on mutual reinforcement with drug and monetary reinforcers resistant to satiation, coupled with the limited effectiveness of any single mitigation strategy, suggests that harm mitigation will be most effective when both consumption and supply are targeted together in a coordinated fashion rather than in isolation. Strategies that target only supply may be effective to reduce one source of supply, but PwSUD deprived of drug reinforcement can be expected to compensate by seeking alternative sources, as occurred during the early stages of the opioid epidemic when individuals with opioid use disorder transitioned from prescription analgesics provided by licit if often unscrupulous medical suppliers to heroin and fentanyl provided by black-market suppliers. Similarly, strategies that target only PwSUD may be effective to reduce consumption by some PwSUD, but suppliers deprived of monetary reinforcement can be expected to compensate by adjusting contingencies of supply (e.g., with lower prices or easier access) or by seeking out new consumers. A coordinated approach would simultaneously and explicitly target both consumption and supply while also anticipating expected changes in behavior by consumers and suppliers.

As a final point, this model of intra- and inter-economon operation may be useful not only to guide strategies for mitigation, but also to investigate, predict, and categorize sources of risk. Thus, mitigation focuses on strategies to impede the operation of a drug-addiction economon, but each node in this model could also be influenced by reciprocal factors that promote their operation. For example, pharmacological antagonists are described here as a pharmacotherapeutic strategy to reduce the intra-economon sensitivity to drug reinforcement, but pharmacogenetic traits (McCorkle et al. 2021) or physiological states (Carr 2002) might enhance it. Similarly, environmental manipulations that enhance access to non-drug alternatives may be useful as inter-economon strategies for mitigation, but reciprocally, environmental circumstances that limit access to non-drug alternatives may enhance behavioral allocation to drug-addiction economa (e.g., Christie 2021).

## Conclusion

An economon is an economic unit composed of two participants engaged in mutually reinforcing behavior. Although many species can form economa that may assemble into rudimentary economies, humans are especially adept at producing a wide array of reinforcing goods and services as potential reinforcers and are also able to use money as a generic token of exchange to facilitate the proliferation of economa. The drug-addiction economon is one type of uniquely human economon in which an addictive drug serves as the reinforcer for one participant, while money usually serves as the reinforcer for the other participant. Drug-addiction economa are especially resilient and transmissible because both drug and monetary reinforcers are resistant to satiation, persistent in their reinforcing efficacy, competitive against other economa, and composed of human behaviors that can be readily taught to or mimicked by new participants. Drug-addiction economa are amenable to both laboratory and field research, and such research may identify new strategies for risk mitigation.
